# Needs assessment survey of healthcare providers’ perceptions and practices regarding delirium prevention at a university medical center

**DOI:** 10.3389/fragi.2022.912142

**Published:** 2022-10-04

**Authors:** Bushra Alghamdi, Siran M. Koroukian, Denise Kresevic, Colin K. Drummond

**Affiliations:** ^1^ Systems Biology and Bioinformatics Graduate Program, School of Medicine, Case Western Reserve University, Cleveland, OH, United States; ^2^ Department of Population and Quantitative Health Sciences, School of Medicine, Case Western Reserve University, Cleveland, OH, United States; ^3^ Department of Nursing, University Hospitals Cleveland Medical Center, Cleveland, OH, United States; ^4^ Department of Biomedical Engineering, Case Western Reserve University, Cleveland, OH, United States

**Keywords:** delirium, survey, assessment, management, prevention, documentation

## Abstract

**Background:** Despite the high prevalence and serious implications of delirium, identification, tracking, and documentation of the condition remain a challenge for the health care team, impeding management of patients. This survey is the first phase of a qualitative study to build a conversational agent-based tool for screening and managing delirium-prone patients.

**Objectives:** To assess healthcare providers’ perceptions of delirium management, focusing on patient assessment, therapeutic interventions, and subsequent communication and documentation.

**Design:** An electronic web-based survey was distributed to healthcare providers identified as caring for inpatient acutely ill older adults admitted for medical and orthopedic surgery needs. Respondent contact information was removed to preserve anonymity.

**Setting:** A 1,000 bed university-affiliated teaching hospital in an urban setting.

**Participants:** 23 residents in family practice, 36 residents in internal medicine, and a total of 492 advanced care nurses, nurses, and clinical staff.

**Approach:** The analysis of survey responses provided insight into providers’ current experiences with delirium assessment tools including computerized documentation, as well as their perceptions and attitudes toward delirium prevention.

**Key results:** Most respondents (89%) thought delirium could be prevented, and 85% thought targeting delirium risk factors was helpful. Fifty one percent reported patients’ loneliness and need for companionship, and 65% believed delirium was linked to higher mortality. Only 14% of respondents thought existing Electronic Health Record (EHR) alerts to identify high-risk delirium patients were useful, and 38% thought current delirium assessment protocols were helpful. In addition, 33% of nurses never received formal delirium prevention training, and 48% indicated that they needed improved systems to assess and manage patients at risk for delirium.

**Conclusion:** A majority of providers affirmed that current delirium protocols are helpful; however, existing screening instruments and methods for documentation are cumbersome, resulting in incomplete or limited documentation of episodes. These barriers lead to an understatement of evidence available for continuous improvement of the patient management process.

## Introduction

Delirium is a common transient neuropsychiatric disorder defined as an acute disorder of attention and cognition. It causes a cascade of negative health outcomes for elderly institutionalized patients (Inouye et al., 1990; Miyagawa et al., 2017). Hospital-acquired delirium is associated with increased morbidity, closer nursing surveillance requirements (less patient independence), higher hospital costs per day, longer hospitalizations, and increased rates of nursing home placement at discharge (Levkoff et al., 1986; Lipowski, 1987). Preventing delirium requires using methods that could potentially lower delirium risk factors and result in better clinical outcomes (Inouye et al., 1999; Mistraletti et al., 2012). Existing evidence shows that delirium is preventable in 30%–40% of cases (Ghaeli et al., 2018).

More recently, delirium was associated with the current novel coronavirus (SARS-CoV-2) pandemic with (Duggan et al., 2021) increased numbers of patients with acute confusion and “brain fog.” Although SARS-CoV-2 affects all ages, adults aged 65 years and older are at the greatest risk of severe disease, hospitalization, intensive care use, and death (Stokes et al., 2020).

To enhance delirium identification, screening instruments have been established and validated in hospitalized and other institutionalized patients, including the Confusion Assessment Method (CAM) (Inouye et al., 1990).

However, integration of these tools into daily practice and electronic health records (EHRs) has been lagging. In addition, several surveys have noted that the current delirium screening tools have poor usability and are not suitable in daily routine clinical screening by nurses because of their length and the required proficiencies to complete them (Inouye et al., 1990; Trzepacz et al., 2001; Shenkin et al., 2018).

CAM is a four-step diagnostic algorithm commonly used as a research instrument for delirium screening. CAM is proven to be the best predictor of increased length of stay and mortality when compared to other delirium screening protocols (Inouye et al., 1990).

The Hospital Elder Life Program (HELP), is a highly effective intervention that relies on volunteers and incorporates core care interventions; this labor-intensive protocol has proven difficult to implement in a number of settings due to staffing shortages. HELP is one of the most used multicomponent protocols targeting delirium risk factors in hospitals and has proven its efficiency in reducing delirium incidents (Inouye et al., 2000; Strijbos et al., 2013). HELP intervention requires many volunteers to deliver protocols aiming at multiple risk factors, including orientation, mobilization, vision, hearing, hydration, nutrition, and sleep. HELP is used in more than 200 hospitals worldwide and serves as the gold-standard nonpharmacological intervention for risk mitigation and delirium management. Additionally, it focuses on cognitive and functional decline, fall prevention, and 1:1 observation of older adults. (Strijbos et al., 2013). While intervention protocols are standardized, assigned interventions are customized to each patient’s abilities and preferences. Interventions are carried out by skilled interdisciplinary teams assisted by trained volunteers. Daily visits, orientation, therapeutic activities, sleep enhancement, early mobilization, vision and hearing adaptation, fluid replacement, and feeding assistance are all included in the program’s core intervention protocols (Hshieh et al., 2018). Additional program interventions include geriatric nursing assessment and intervention, interdisciplinary rounds, ongoing staff education, post-discharge community connections, and telephone follow-up (Hshieh et al., 2018).

Although the HELP program is cost-effective in the care of high risk populations, it requires substantial training and monitoring of a large team of volunteers, which can serve as a barrier to adoption, especially with limited resources (Rubin et al., 2006; Caplan and Harper, 2007). Further, the availability of professional caregivers to continually manage high-risk patients for delirium is not always ensured, thus impacting continuity and congruency of data.

## Methods

### Setting and survey design

The institutional review board (IRB) determined that this study fits the criteria for exemption from IRB review and approved it (the study number: STUDY20210175). The study was conducted in a university-affiliated teaching hospital in an urban setting. Our study site has been recognized as an exemplar which is the highest and most prestigious level of recognition by Rory Meyers College of Nursing through their Nurses Improving Care for Health System Elders (NICHE) program. Current screening is conducted by nurses using CAM assessments, and the score is discussed with MD physicians during rounds. In addition, they identified age-friendly level 2 commitment to care, which is a geriatric recognition indicating an ongoing assessment of cognition. The scores are documented using nursing flowsheets in the Electronic Medical Record (EMR).

For the distribution and management of survey data, we used REDCap (Research Electronic Data Capture), a secure, web-based software platform designed to support data capture for research studies (Harris et al., 2019). We designed and distributed a survey with a Likert scale, yes/no, and multiple-choice questions through a secure link from REDCap to healthcare providers’ email addresses. We recruited clinicians who had been identified as having an interest in the care of older adults at medical, surgical, and ICU units. These units and their head nurses expressed an interest in delirium prevention. An email list of all nurses was sent from the Chief Nursing Officer (CNO).

This survey was piloted with a panel of seven clinical experts in delirium and sent to healthcare providers *via* email. We used the following 2-step process: First, a general, basic email inviting people to click a link for more information. Then, once an individual clicked on the link to enter the survey, a full information sheet appeared before the survey itself.

### Data collection

Participation in the survey was voluntary and according to the study protocol they could discontinue at any time. The survey did not collect any identifiable information, so it was anonymous. Consent was implied by completion of the survey and a written explanation of the research was provided. All data were aggregated and collected in the REDCap system.

### Data analysis

We imported the data from REDcap and readied it for analysis in R. Herein, we report our findings from our descriptive analysis based on frequencies and percentages using contingency tables, Likert scale and binary variables.

## Results

### Participants’ characteristics

A total of 97 surveys were collected from a pool of 575 healthcare providers, full and part time employees, with a response rate of 17%.

The majority of participants (49%) were nurses, followed by 21% Physicians, and 12% clinical support staff (Table 1).

**TABLE 1:** Sample characteristics.

Characteristic	*N* = 97
Healthcare provider title	
Physician (MD or DO)	20 (21%)
Advanced Practice Nurse (NP, CNS)	17 (18%)
Nurses	47 (49%)
Other (Clinical support Staff)	11 (12%)
Unknown	2

### Healthcare providers’ perceptions and current practices

Healthcare providers’ perceptions of delirium were assessed through their attitudes toward delirium prevention protocols and their assessment of what they find useful. When asked how frequently they document delirium in the EHR, the majority (51%) confirmed that they do so. Additionally, they believed that the majority of delirium episodes could be prevented, as shown in Table 2. The healthcare team’s beliefs regarding the use of current evidence-based protocols strongly suggest their effectiveness with an overall positive response rate of 86 percent (38 %Yes, 48% Sometimes). Eighty-five percent of respondents agreed that it is important to manage delirium risk factors; however, 28% reported not documenting delirium in the EHR, and 22% reported documenting it infrequently (Table 2).

**TABLE 2:** Reported beliefs in delirium screening.

Statements	*N* = 97
Do you always document delirium in the EHR?	
No	27 (28%)
Yes	49 (51%)
Sometimes	21 (22%)
Do you think the NICHE, HELP, ACE, protocols are helpful to manage delirium known risk factors?	
No	13 (14%)
Yes	35 (38%)
Sometimes	44 (48%)
Unknown	5
Do you think it’s helpful to manage delirium known risk factors?	
No	2 (2.6%)
Yes	66 (85%)
Sometimes	10 (13%)
Unknown	19
Many cases of delirium can be prevented	
No	10 (11%)
Yes	83 (89%)
Unknown	4

**FIGURE 1:**
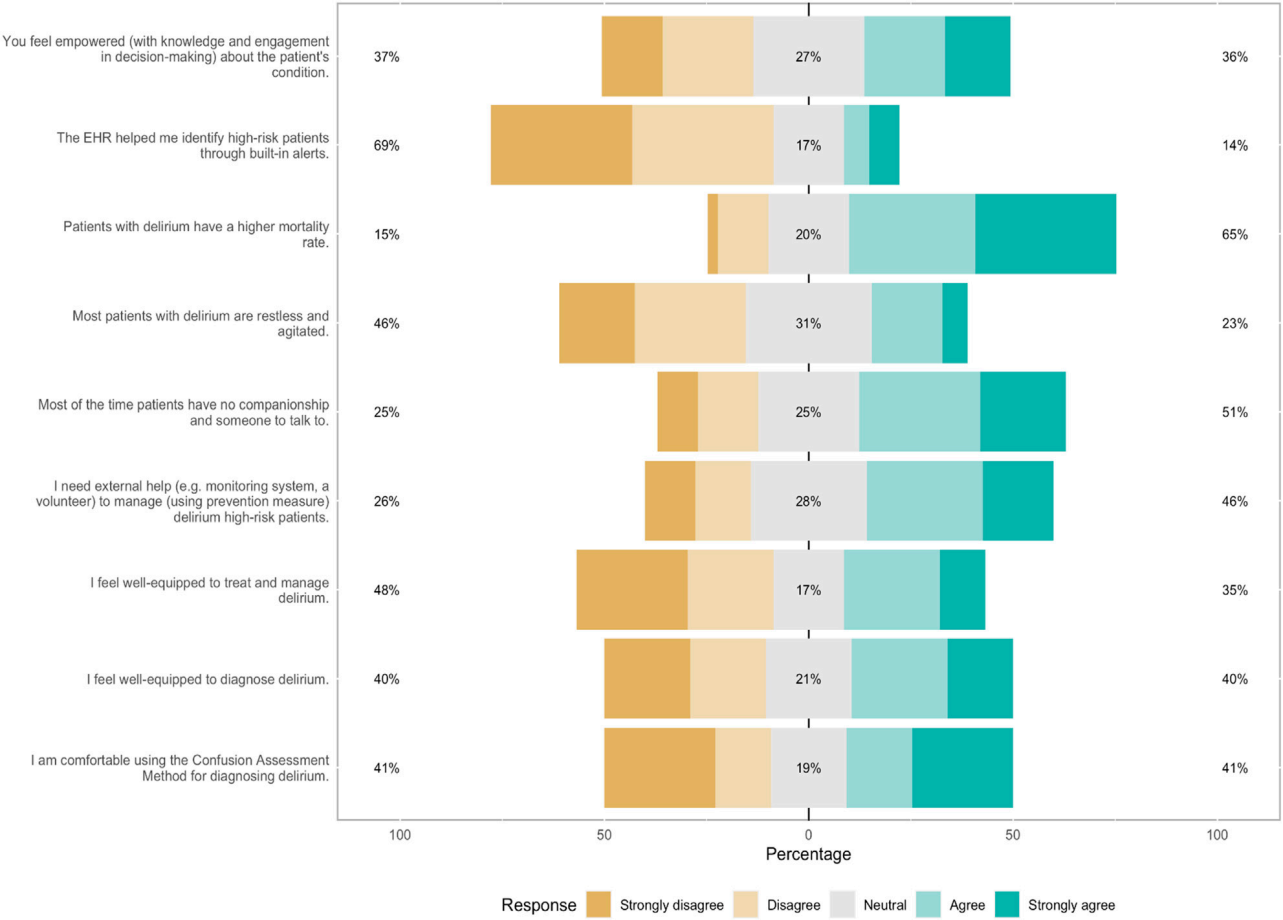
Likert scale of healthcare professionals of delirium current practices and beliefs.

In this study, while the majority of participants (69%, *n* = 90 strongly disagreed that EHR helped them identify high-risk patients of delirium through built-in alerts, 41% (*n* = 94) reported not screening with the Confusion Assessment Method (CAM) which is the current standard screening instrument (Figure 1). Forty-six percent strongly agreed when asked about their need for external help (monitoring system and volunteers) to manage delirium in high-risk patients, and 46% indicated that delirious patients were agitated and restless (Figure 1).

### Delirium education

**FIGURE 2:**
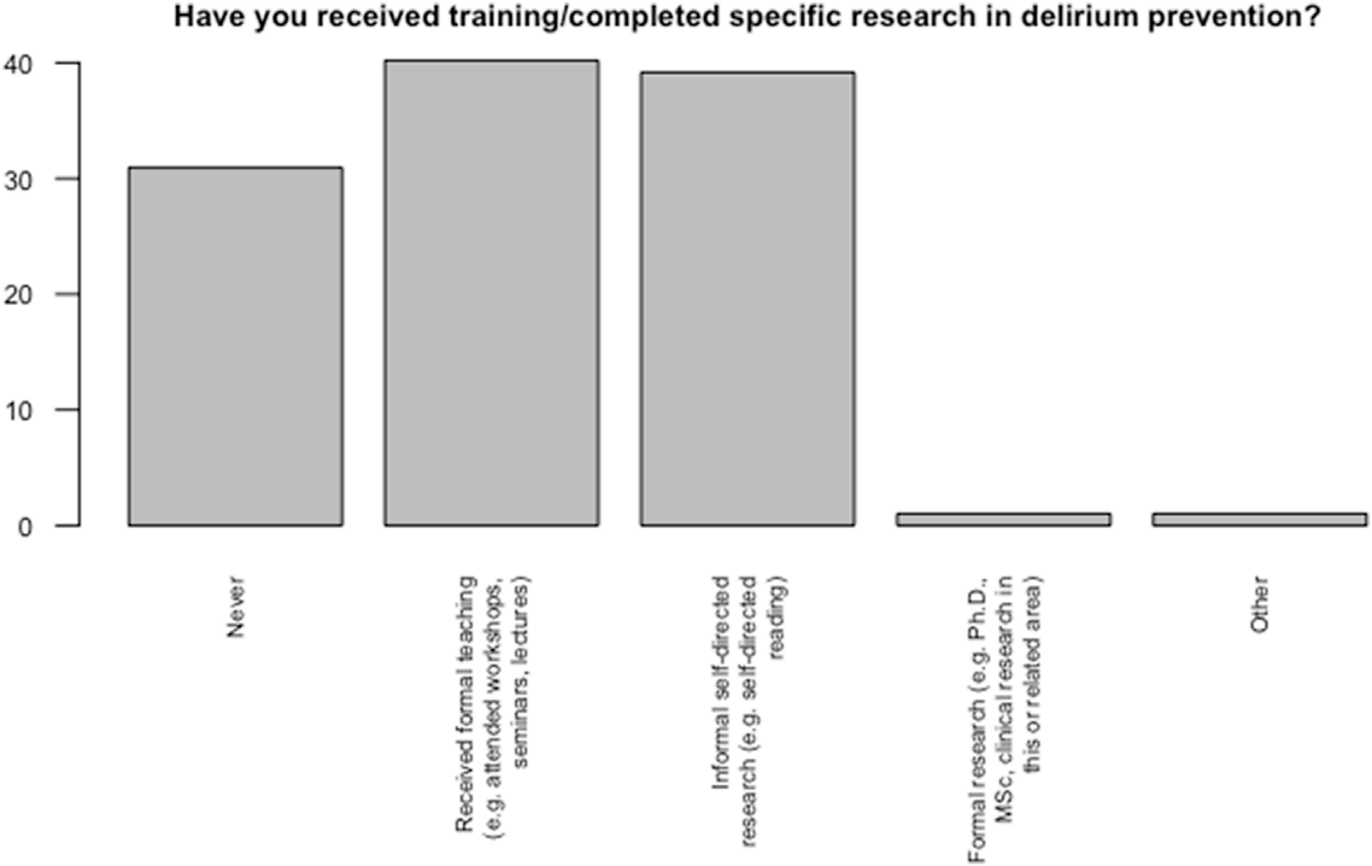
Healthcare providers’ training pertaining to delirium prevention.

Our findings indicated that 30 (32.6%) of healthcare providers had never received any formal or informal training pertaining to delirium prevention. On the other hand, 39 (42.4%) indicated receiving formal teaching (e.g., attended workshops, seminars, lectures) and 38 (42.4%), received informal self-directed research (e.g., self-directed reading) (Figure 2).

**FIGURE 3:**
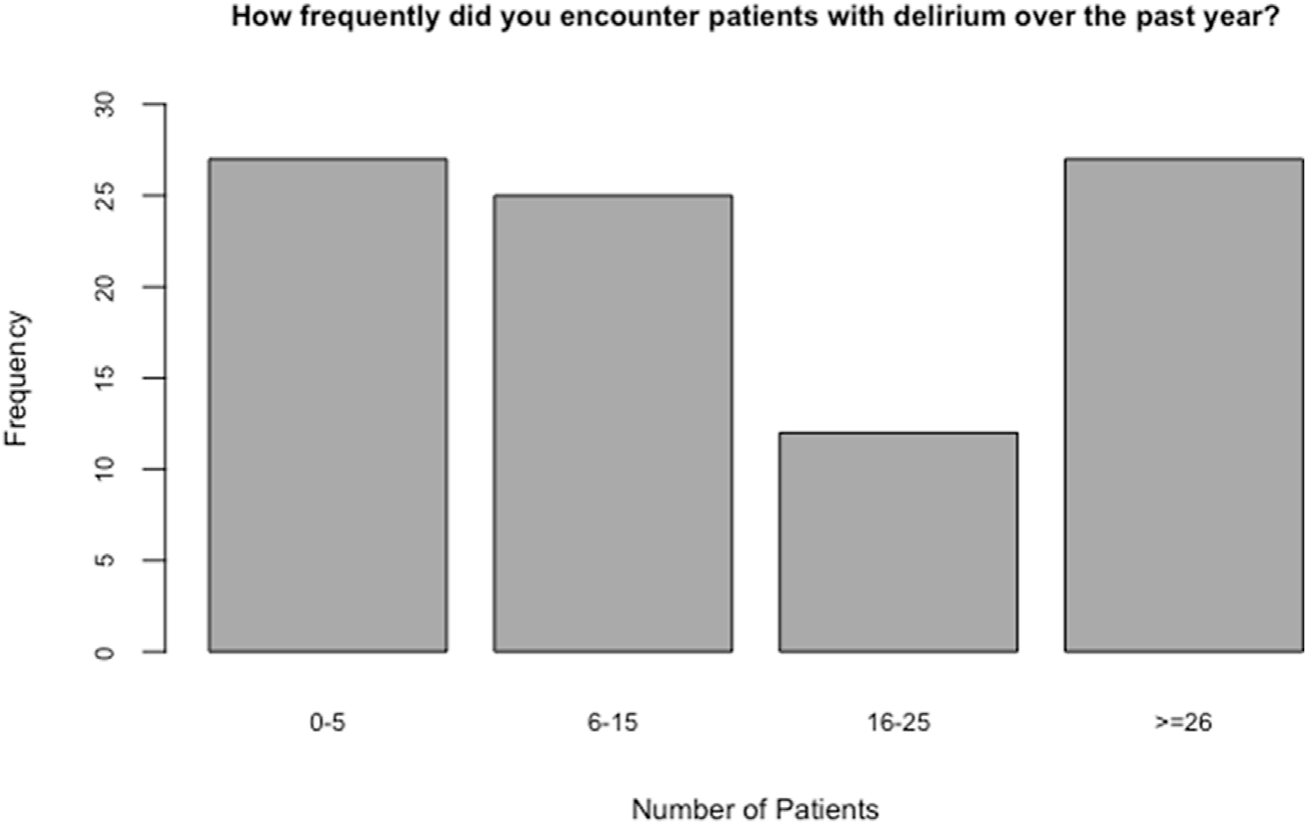
Delirium patients’ seen by healthcare providers in a year (frequency of delirium episodes).

Data indicates that 46 nurses (41.2% of nurses) had received formal education and training on delirium prevention and 46 (39.1%) (*n* = 46) had informal type of education (Table 3). Thirty percent of healthcare providers reported seeing more than 26 patients with delirium over a year period (Figure 3).

**TABLE 3:** Delirium prevention education/training reported by healthcare providers categorized by type of healthcare provider.

Healthcare provider title	Delirium prevention education/Training received by healthcare providers					
	Never	Received formal teaching (e.g., attended workshops, seminars, lectures)	Informal self-directed research (e.g. self-directed reading)	Formal research (e.g., Ph.D., MSc, clinical research in this or related area)	Other training	Total
Physician (MD or DO)	7 (38.9%)	7 (38.9%)	8 (44.4%)	0 (0.0%)	0 (0.0%)	18 (100.0%)
Advanced Practice Nurse (NP, CNS)	4 (23.5%)	9 (52.9%)	8 (47.1%)	0 (0.0%)	0 (0.0%)	17 (100.0%)
Nurse	15 (32.6%)	19 (41.3%)	18 (39.1%)	1 (2.2%)	1 (9.1%)	46 (100.0%)
Other (Clinical staff)	4 (36.4%)	4 (36.4%)	4 (36.4%)	0 (0.0%)	1 (9.1%)	11 (100.0%)
Total	30 (32.6%)	39 (42.4%)	38 (41.3%)	1 (1.1%)	1 (1.1%)	92 (100.0%)

## Discussion

This is a two-phase study. The first phase is a qualitative study that includes surveys and focus groups as a preliminary step toward defining the need for automating the delirium management protocol. This survey conveyed the needed baseline for our system development phase to understand the current practices and beliefs of the care team with delirium management at this tertiary care center.

Assessment of delirium is difficult. Hyperactive delirium is usually identified by the patient’s abnormal activity and agitation. In contrast, hypoactive delirium is difficult to assess because patients may not have obvious behavioral issues and agitations (Inouye et al., 2001). Sixty-nine percent of respondents believed that delirium was associated with increased mortality rates, and forty-six percent of respondents strongly disagreed that delirium patients are primarily agitated, indicating that the hypoactive subtype is the most common. Numerous studies support the association between delirium during hospitalization and poor outcomes, including mortality (Witlox et al., 2010; MacLullich and Hall, 2011; Krogseth et al., 2014; Krogseth et al., 2014; Marcantonio, 2017; Oh et al., 2017). Our survey results revealed that healthcare providers encountered delirium patients very frequently in their practice. However, 32.6% had never received any training in delirium prevention.

Advances in artificial intelligence technologies such as machine learning and speech recognition systems are believed to have the potential to add value to delirium management since these tools can automate parts of the assessment process, thus reducing staff burden for protocol implementation. In particular, speech recognition methods have rapidly advanced over the past few years in the field of voice analytics for assessing and managing patients with mental illness. Speech recognition is believed to be affordable, noninvasive, and simple to deploy remotely, all of which may contribute to improved delirium assessment and management.

It is commonly established that healthcare providers’ perceptions and attitudes concerning delirium are critical to the widespread use and sustained uptake of interventions and evaluation techniques for patient care. With limited published literature available on provider perception issues in delirium care, we established a two-phase research effort to elucidate perception issues. The first phase involved a qualitative study to assess existing delirium knowledge, attitudes, and practices, as well as current needs and barriers to delirium management. This first phase had two complementary activities. First, we conducted a survey to establish a baseline dataset for a tertiary care center that provides geriatric care. Following the survey, we conducted focus group activity to provide more granularity to perceptions of providers on the challenges and explore more in-depth information to address barriers.

The second phase of the research program integrates the qualitative focus group results and the quantitative survey results that is then used in support of the specification development for a delirium management system utilizing a voice recognition technology.

The current work reports on the characterization of providers’ use of delirium screening protocols and to understand their perceptions of delirium prevention as well as its current challenges. Our purpose was to improve our understanding of needs for the development of an automated delirium assessment and management system (ADAMS) designed to overcome the unstandardized method of screening for delirium and the variability of training and experience of the clinical staff. Our system is complementary to nurses’ care and intended to improve the current time-intensive frequent bedside assessment protocols. ADAMS will automate the most successful delirium prevention protocols and assessments like HELP and CAM using voice recognition system device at the bedside near the patient. The system will include prompts, reminders, and alert messages to ensure efficient communication between the healthcare provider team and the patient. As well as documenting the interaction between the patient and the system in the EMR.

Conventional delirium prevention strategies emphasize increased social interaction which is very limited given the current nursing staff shortage. In addition, this shortage was exacerbated recently by the pandemic which imposed isolation for most patients and families. Delivering these types of treatment such as HELP program and volunteers was more difficult in COVID-19 settings, where the patients required isolation. Therefore, our ADAMS intervention could provide valuable and needed companionship to the patients and their families. The proposed ADAMS would screen for early warning signs of delirium and will focus on four delirium-prevention strategies: orientation, mobility, sleep, pain evaluation, and nutrition.

This study provided valuable insights. However, several limitations must also be acknowledged. Self-reporting responses will invariably contain inaccuracies due to response bias, which may be a result of the poor recall of clinical experiences or misinterpretation of questions. The responses may have been influenced by the culture of the study site, which may limit the study’s generalizability. Also, The survey was conducted during the COVID-19 pandemic, which was associated with increased delirium incidence (Aguilar-Navarro et al., 2020), and which may have influenced the results of the survey. As reported elsewhere, the dramatic increase in delirium incidence in patients admitted with COVID-19 has been associated with extended lengths of stay and isolation (Stokes et al., 2020).

In conclusion, while the majority of responders recognized delirium as a serious problem, 30% denied having received training that may have contributed to their lack of knowledge regarding assessment and management of delirium. According to the survey, barriers to caring for patients with delirium exist. Screening instruments are not perceived as easy to use. Documentation is inconsistent, potentially decreasing communication to the health care team and delaying treatments. Current interventions are also difficult to implement given staffing shortages. Strategies to improve care will need to address these caregiver issues. Technological advances such as conversational agents may help provide streaming data to caregivers in an efficient manner. These findings support the need to develop ADAMS a system that combines delirium prevention and screening protocols to provide early warning system and conversational agent support for enhanced patient/caregiver interaction.

Given the results of this survey that noted participants viewed delirium as a serious condition it appears they may be supportive of efforts to enhance education and documentation of this condition. These findings provide an opportunity to enhance our care of delirious patients.

Historically education regarding delirium prevention, assessment, and management have been taught using didactic lectures. However, emerging evidence continues to support the need to do multi-modal education including the use of case studies and simulation (Inouye et al., 2001; Middle and Miklancie, 2015). In summary, there is a demand for a more standardized, usable protocol to assist in screening and management of hospital-acquired delirium.

## Conclusion

In conclusion, most nurses and physicians consider delirium to be a serious problem and encounter it very often. However, current delirium screening tools and documentation systems are inefficient which leads to missed opportunities to prevent delirium or at the very least intervene early to prevent further cognitive and functional decline in frail older acutely ill hospitalized patients. This leads to poor delirium monitoring for the geriatric population. The survey provides support for further study of delirium assessments, documentation, communications, and management systems that could aid bedside care givers. A standardized interactive computerized system to improve the present healthcare providers’ assessments and management of high-risk delirium patients is a key unmet need. Capturing bedside caregivers’ perspectives is essential to building accurate and efficient software programs to increase identification, communication, and management of patients with delirium. Increasing numbers of older adults and the continued health care staffing shortage necessitate the need to leverage technological advances to enhance care. Future work is to convene focus groups to validate and further explore the findings of our survey.

## Data Availability

The raw data supporting the conclusion of this article will be made available by the authors, without undue reservation.
